# Research Progress on Strategies for Improving the Enzyme Properties of Bacteriophage Endolysins

**DOI:** 10.4014/jmb.2312.12050

**Published:** 2024-02-28

**Authors:** Yulu Wang, Xue Wang, Xin Liu, Bokun Lin

**Affiliations:** 1 Shunde Women and Children's Hospital, Guangdong Medical University, Foshan 528300, P.R. China; 2 Dongguan Key Laboratory of Public Health Laboratory Science, School of Public Health, Guangdong Medical University, Dongguan 523808, P.R. China

**Keywords:** Phage, endolysin, bactericidal activity, lysis spectrum, outer membrane permeabilizers

## Abstract

Bacterial resistance to commonly used antibiotics is one of the major challenges to be solved today. Bacteriophage endolysins (Lysins) have become a hot research topic as a new class of antibacterial agents. They have promising applications in bacterial infection prevention and control in multiple fields, such as livestock and poultry farming, food safety, clinical medicine and pathogen detection. However, many phage endolysins display low bactericidal activities, short half-life and narrow lytic spectrums. Therefore, some methods have been used to improve the enzyme properties (bactericidal activity, lysis spectrum, stability and targeting the substrate, etc) of bacteriophage endolysins, including deletion or addition of domains, DNA mutagenesis, chimerization of domains, fusion to the membrane-penetrating peptides, fusion with domains targeting outer membrane transport systems, encapsulation, the usage of outer membrane permeabilizers. In this review, research progress on the strategies for improving their enzyme properties are systematically presented, with a view to provide references for the development of lysins with excellent performances.

## Introduction

Bacteriophages are viruses that specifically invade and infect bacteria. They are divided into temperate phages and virulent phages. Virulent phages could proliferate rapidly in bacterial cells, produce endolysins to lyse the bacteria and release offspring phages [1, 2]. Phage endolysins are known as peptidoglycan-degrading proteins that could rapidly induce the lysis and death of bacteria by targeting the chemical bonds of peptidoglycan (PG) on the bacterial cell wall [3]. Because these PG hydrolases lyse ‘‘from within’’, they are referred to as ‘‘endolysins’’ or simply ‘‘lysins’’ [4].

**Fig. 1:**
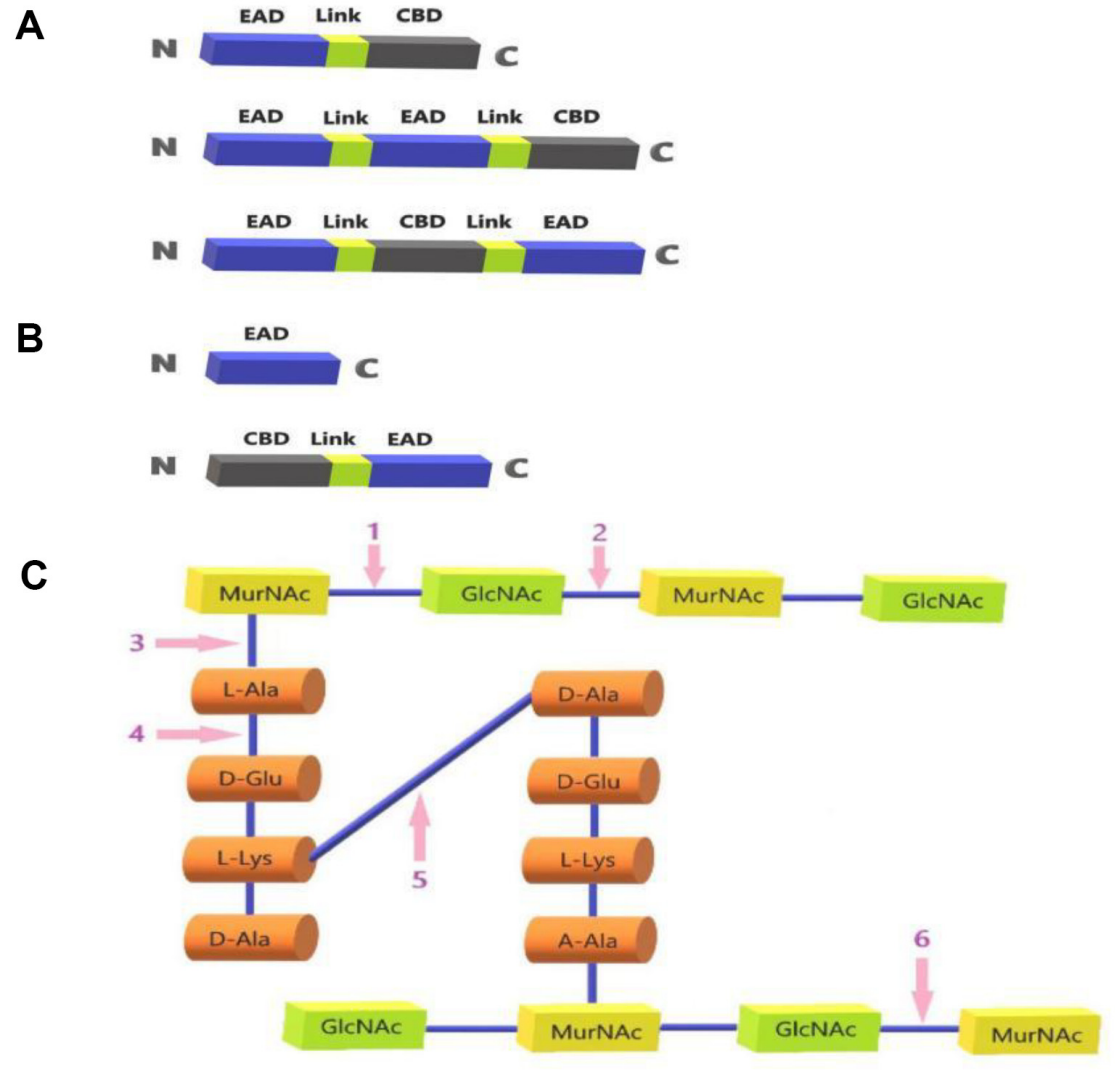
Structures of bacteriophage lysins and their catalytic sites. (**A**) Basic structures of GP-Lysins; (**B**) Basic structures of GN-Lysins; (**C**) Catalytic sites of phage lysins (1: N-acetyl cyclase; 2: N-acetyl-β-D-aminoglucosidase; 3: Nacetylcytidyl-L-alanine amidase; 4: peptide chain endonuclease; 5: peptidase; 6: transglycosidase); where: GlcNAc: Nacetylglucosamine; MurNAc: N-acetylcytidylic acid; L-Ala: L-alanine; D-Glu: D-glutamic acid; L-Lys: L-lysine; D-Ala: Dalanine; L-Gly: L-glycine.

According to the Gram-staining of host bacteria, bacteriophage endolysins are divided into two groups. One could be called GP-Lysins which are produced by bacteriophages of Gram-positive bacteria (GP-phages). The other one could be called GN-Lysins which are produced by bacteriophages of Gram-negative bacteria (GN-phages). GP-Lysins usually have a modular structure. As shown in Fig. 1A, GP-Lysins contain two domains, namely the N-terminal catalytic domain (EAD) and the C-terminal cell wall binding domain (CBD), which are connected by a short peptide. The EAD is capable of acting on most chemical bonds of the PG network in the bacterial cell wall to cause bacterial lysis [1], while the CBD is responsible for targeting the endolysin to the substrate and conferring specificity for recognizing host cells. The high specificity of lysins offers it an advantage over conventional antibiotics as endolysins do not disturb the normal microflora. Typically, these is a flexible interdomain linker sequence between the EAD and the CBD [5]. Usually, endolysins have only one EAD and one CBD, but some lysins were found to have more than one CBD or EAD arranged in different order [6-8].

As shown in Fig. 1B, GN-lysins usually only have one globular structure with a single EAD [9, 10]. Gram-negative bacteria have an outer membrane (OM) composed of lipopolysaccharide (LPS) compared to Gram-positive bacteria. The OM of Gram-negative bacteria effectively prevents GN-lysins from acting on the cell wall externally. To date, only a few GN-lysins are able to lyse Gram-negative bacteria without the help of OM permeants. These GN-lysins contain amphipathic helical structures or carry positively charged groups which confer the ability to penetrate or disrupt the bacterial OM, thereby accessing and degrading the PG layer and ultimately leading to bacterial lysis and death [11, 12]. For example, the α-helical structure formed by the C-terminus of lysin AcLys enables it to penetrate the OM. Additionally, the positively charged groups present in the C-terminus enhance its ability to penetrate the OM [11, 13]. However, there are also a few of GN-Lysins owning a modular structure with a CBD at the N-terminus and a EAD at the C-terminus [14, 15]. These GN-Lysins obtain high lytic activities towards Gram-negative bacteria due to the presence of CBD which helps the lysins get close to the substrate [14, 16]. Different endolysins differ considerably in protein structure and enzymatic activity. Usually, the endolysin exhibits only one hydrolytic activity. As shown in Fig. 1C and Table 1, depending on the site of action of the EAD on the bacterial cell wall, lysins can be classified into four categories [17]. They are N-acetylmuramidase, transglycosylase, amidase and endopeptidase.

Many GP-lysins exhibit rapid "contact-dependent" bactericidal activity, capable of reducing bacteria to undetectable levels within seconds [25, 26]. Comparatively, the bacterial killing kinetics of GN-lysins are in a slower manner. Endolysins contain at least one structural domain responsible for the enzymatic cleavage of PG, also known as murein, which is the major structural component of the bacterial cell wall. PGs form a vesicle-like structure that surrounds the bacterial cytoplasmic membrane and imparts the necessary mechanical resistance to avoid cell lysis [27]. Therefore, uncontrolled breakdown of the murein structure typically results in osmotic cell lysis. Significantly, exogenous addition of endolysins to a susceptible host can be exploited to produce lysis from without due to the high osmotic pressure within the cell [28]. In 2001, Nelson *et al*. first demonstrated that endolysins could be used as antibacterial agents to treat related diseases [3]. Since then, researchers had paid increasing attention to the study of endolysins. In addition, the excessive and abusive use of antibiotics has led to increasing bacterial resistance to antibiotics [29]. And the development of new methods that can effectively combat bacterial infections has become an imminent task. Compared to traditional antibiotics, endolysins have advantages such as high bactericidal specificity, not altering the normal flora, low probability of inducing bacterial resistance, can be genetically engineered, having synergistic effects with other antibacterial agents, effective elimination of biofilm and easy accessibility [30-33]. However, many native endolysins displayed low bactericidal activities and other defects. Therefore, some methods have been developed to improve the bactericidal activity and stability of endolysins. This review systematically describes the research progress on strategies for improving enzyme properties of endolysins.

## Strategies for Improving Enzyme Properties of Endolysins

GP-Lysins usually show good antibacterial activities against Gram-positive bacteria. They generally have a modular structure, consisting of multiple EADs and CBD, which can be used as the basis for modifying the functional domains, such as deletion or addition or direct mutation of domains, chimeolysins and so on to improve the bactericidal activity, bactericidal spectrum, stability, solubility and adaptability to the environment.

The OM of Gram-negative bacteria effectively prevents most lysins from reaching the PG layer and acting on the cell wall externally. Though there were a few of GN-Lysins reported to have the ability to penetrate the OM by themselves, it should be noted that these lysins often suffer from limited availability and insufficient cleavage activity [11, 12, 34, 35]. Therefore, how lysins can pass through the OM of Gram-negative bacteria become a key point. Here in this review, the methods that can improve the activity of lysins against Gram-negative bacteria are summarized including fusion with the membrane-penetrating peptides (MPPs), fusion with domains targeting OM receptors or transport systems, encapsulation strategy and using OM-penetrating agents. The methods used to improve GP-Lysins are also applicable to GN-Lysins and vice versa. It is also possible to explore the development of lysins with excellent performances by interoperability of multiple methods.

### Deletion of Cell Wall-Binding Domain or Changing the Net Charge of Catalytic Domain

Classical lysins require a CBD that targets the catalytic domain to the PG layer. For these classical lysins, specificity and bacteriolytic activity require the strong binding of the CBD to the cell wall. Once the CBD was deleted, the catalytic domain would lose lytic activity. Therefore, these classical lysins are CBD-dependent. However, some lysins are CBD-independent and the removal of the CBD conversely expanded the lytic spectrum and increased the lytic activity. For example, the truncated lysin PlyGBS90-1 consisting of the EAD and the last 13 amino acids at the C-terminal end of the wild lysin PlyGBS obtained a 28-fold higher lytic activity against group B *Streptococci* than the full-length enzyme [36]. The C-terminal truncated protein retaining the EAD of lysin CD27L obtained an increased lytic activity against *Clostridium difficile* and a broadened lytic spectrum whereas the N-terminal truncated protein had no lytic activity [37]. It was found that the removal of CBD of CBD-independent lysins could alter the net charge of EAD to be positive [43]. Since the cell wall of Gram-positive bacteria is usually negatively charged. Therefore, the removal of CBD of CBD-independent lysins may lead to increased interactions between the EAD and the cell wall. That is why the CBD-independent lysins obtained an increased lytic activity when their CBDs were deleted. On the other hand, the CBD-dependence of lysin could be eliminated or created by engineering a reversal of sign of the net charge of the EAD [43]. Therefore, deletion of CBD or changing the net charge of EAD could be a facile approach for improving the lytic activity of CBD-independent lysins.

### Addition of Domains

Increasing the affinity of endolysins to target cells by modifying the CBDs could improve the lytic efficacy. Schmelcher *et al*. demonstrated that the endolysin Ply500 equipped with an extra copy of its natural CBD improved the affinity by 50-fold and increased its ability to act at high salt concentrations [38]. The endolysin HydH5SH3b was generated by the addition of lysostaphin CBD (SH3b) to lysin HydH5 of *Staphylococcus aureus* phage phiIPLA88, had a higher activity and thermostability than the parental HydH5 [39]. The possible reasons were that lysins in solution may require the CBD to optimally recognize their substrate and the extended lysin HydH5SH3b may have a higher conformational stability than the wild type lysin HydH5. Although CBD is not always necessary for basic activities of lysins, it is needed to achieve high levels of activity for some lysins.

### DNA Mutagenesis

Various DNA mutagenesis methods including site-directed mutation and random mutagenesis, had been used to produce mutants of lysins with enhanced lytic activity and/or stronger thermostability. The mutation of 15 amino acids in the CBD of the pneumococcal lysin Cpl-7, resulted in the inversion of the sign of the charge of CBD, was performed to generate the mutant lysin Cpl-7S which obtained increased lytic activities and an expanded lytic spectrum against *Streptococcus pneumoniae*, *S. pyogenes* and other pathogens [40]. These findings provided a strategy to improve the lytic activity of endolysins based on facilitating their pass through the negatively charged bacterial envelope by modulating the net charge of the CBDs [40]. The 88^th^ amino acid glutamate of lysin LysF1 was not conserved and was mutated into three hydrophobic amino acids to generate three mutants, namely Glu88Leu, Glu88Phe and Glu88Met, respectively [41]. Compared to wild lysins, these three mutants obtained improved thermal stability and lytic activity. Endolysin CD27L caused cell lysis of the pathogen *C. difficile*. The Leu 98 of lysin CD27L was modified to a Trp residue which was found in an endolysin from a bacteriophage of *Listeria monocytogenes* (PlyPSA). This mutation in CD27L resulted in an increased activity against selected serotypes of *L. monocytogenes*, implying that the catalytic domain alone contained features that target a specific bacterial species and demonstrating the potential to tune the species specificity of the catalytic domain of an endolysin [37]. With the development of gene synthesis technology, it has become easier to perform point mutations on genes. However, the selection of amino acid residue for the point mutation remains a great challenge.

### Chimerization of Domains

The domains of endolysins can be exchanged or recombined with other domains to generate chimeolysins with desirable properties. This approach could increase the catalytic capacity of the EAD and/or the ability of the CBD to recognize substrates. Especially, the substitution of CBD is the most commonly used means. New CBDs could alter the bactericidal activity and host specificity of the chimeric lysin. Chimeolysins usually could obtain broader lytic spectrums, higher lytic activities or other improvements. To further improve the lytic activity, Ply187AN was fused with the CBD of lysin LysK to generate the chimeolysin Ply187AN-KSH3b which was more active against *S. aureus* than Ply187AN [42]. The chimeolysin Ply187N-V12C which was produced by the fusion of the EAD of staphylococcal lysin Ply187 and the CBD of enterococcal lysin PlyV12 obtained an extended lytic spectrum including not just *Staphylococcus* but also *Streptococcus* and *Enterococcus* [43]. The CBD of the staphylococcal lysin Lys87 was recombined with the EAD of enterococcal lysin Lys168 and Lys170 to generate the chimeric lysin Lys168-87 and Lys170-87, respectively [44]. These two chimeolysins exhibited a broader lytic spectrum against *Enterococci*, *Streptococci* and more than 96% of tested clinical isolates of *S. aureus*. Their solubilities were also enhanced.

Chimeolysins compensate for the deficiencies of single lysins and avoid degradation of exogenous gene expression products by the host cell protease system. It has been shown that chimerization of lysins with similar hosts may broaden their lytic spectrums and chimerization of lysins with different hosts may change and broaden their efficacy in a wide range of genera [10].

### Fusion to the Membrane-Penetrating Peptides

The main component of the OM of Gram-negative bacteria is LPS of which the stability is maintained by the ionic interactions between divalent cations and phosphate groups and the hydrophobic accumulation of lipid A. The majority of these MPP have a positive net charge, but there were also anionic antimicrobial peptides [45]. These peptides possess both hydrophilic and hydrophobic regions on their surfaces. The cationic section of the peptide interacts with the negatively charged bacterial cell surface through electrostatic interactions, while the hydrophobic section interacts with the lipids present in the bacterial membrane. As a consequence, the MPP could help the lysin to pass through the LPS layer, promote a change in the OM and reach the periplasmic space and degrade the PG leading to the eventual death of the bacteria [46, 47]. In a 2019 study, it was found that the fusion of hydrophobic amino acids at the C-terminus of the lysin Lysep3 also increased antimicrobial activity against *E. coli* and the lytic activity was positive related to the number of hydrophobic amino acids within a certain range [49].

Therefore, MPP plays a key role in this strategy for enhancing the lysins’ activities. In an early study, seven MPPs were screened for fusions with two modular lysins (Lysin OBPgp279 from *Pseudomonas fluorescens* phage; Lysin PVP-SE1gp146 from *Salmonella* phage) at the N-terminal, respectively [48]. Within these 14 recombinants, lysins LoGT-001 and LoGT-008 carrying the membrane-penetrating peptide PCNP (Amino acid sequence: KRKKKRKKKKRK) obtained enhanced antibacterial activities against *P. aeruginosa* and *A. baumannii*. The fused lysin with higher bactericidal activity towards *E. coli* was LoGT-037, in which the two MPPs of PCNP and HPP (hydrophobic pentapeptide FFVAP) were tandemly fused to the N-terminus of lysin OBPgp279. Since MPPs tend to penetrate the OM of a wide range of bacteria, lysins fused with MPPs usually obtain a broader lysis spectrum than the wild enzyme. It was reported that lysin Lyt mu 1/6 lysing *Streptomyces aureus* was fused with a cationic MPP of lysozyme T4 to generate an artificial lysin LytAmfi obtaining a broader lytic spectrum than the wild lysin, with lytic activities against a wide range of clinically common Gram-negative bacteria including *E. coli*, *Bacillus immobilis* and *Citrobacter fumigatus* [50].

There is no fixed pattern for the fusion of the MPP with lysins. The fusion position may be the N-terminal or C-terminal end or both the two ends of the lysin. The best fusion pattern for different lysins should be explored experimentally. Fusion with MPP is not only applicable to GN-Lysins but also to GP-Lysins.

### Fusion with Domains Targeting OM Receptors or Transport Systems

Bacteriocins are antimicrobial peptides or proteins produced by bacteria that could inhibit or kill the closely related bacteria [51]. Bacteriocins can act by targeting specific receptors on the surface of susceptible bacteria, leading to the disruption of membrane integrity and subsequent cell death [52, 53]. The fusion protein of lysin and bacteriocin could exploit the delivery systems used by bacteriocins to translocate through the OM and reach the periplasm to induce PG cleavage [54]. For example, pyocin S2 (PyS2), which is a bacteriocin of *P. aeruginosa*, was fused to the GN4 lysin to generate the PyS2-GN4 lysocin, which could pass through the FpvAI protein channel in the bacterial OM by active transport and enter the periplasm and disrupt the PG layer leading to intracellular membrane imbalance and cytoplasmic leakage [54].

The OM transporter protein of bacteria could also help the lysin to cross the OM of the target bacteria and reach the PG layer. Pesticin is a bacteriocin produced by *Yersinia pestis*. The binding domain of pesticin specifically targets the OM transporter FyuA which is responsible for the toxin uptake and common in pathogenic bacteria. The binding domain of pesticin was fused with the N-terminus of *E. coli* phage T4 lysozyme to form a hybrid lysin [55]. This hybrid lysin kills specific *Yersinia* and pathogenic *E. coli* strains and, importantly, can evade the pesticin immunity protein (Pim) giving it a distinct advantage over pesticin. An analogous approach was used to promote OM translocation of Lysep3, the endolysin of *E. coli* phage *vB_EcoM-ep3*. The translocation and receptor binding domains of colicin A were fused with lysin Lysep3 to construct a fusion lysin Colicin-Lysep3 targeting the receptor BtuB on the OM of *E. coli*, which showed good bactericidal activities against *E. coli* both in vivo and in vitro. It was demonstrated for the first time that colicin A fragment could enable the lysin to lyse *E. coli* externally [56].

Moreover, the fusion of the receptor-binding proteins (RBPs) with endolysins, coined as “Innolysin”, has recently been introduced as a novel approach to target Gram-negative bacteria [57]. Phages specifically recognize bacterial surface receptors through RBPs. The Pb5 monomer located in the tail of phage T5 is a good RBP that specifically and stably binds to the bacterial receptor FhuA [58]. The RBP Pb5 was fused with 23 phage endolysins to construct 228 novel RBP-endolysin hybrids. Among these innolysins, the innolysin Ec21 which was fused by the endolysin of phage T5 with RBP Pb5 had the highest antibacterial activity reducing *E. coli* by 2.20 ± 0.09 log [57]. This innolysin approach requires the presence of a corresponding receptor on the OM of Gram-negative bacteria.

### Encapsulation Strategy

In addition to the fusion engineering approach, GP-lysins can be encapsulated to enhance their stabilities and permeabilities. Liposomes have spherical structures composed of one or more phospholipid bilayers with a core of water [59]. It is safe for the human body and is therefore widely used to deliver proteins, enzymes, vitamins and antioxidants [60]. Liposomes are known to be able to penetrate bacterial membranes by membrane fusion. Depending on the surface charge of the target site, liposomes can be prepared in cationic or anionic form. Most Gram-negative bacterial membranes have an anionic surface and cationic liposomes have a higher antibacterial effect on Gram-negative bacteria than anionic liposomes because of the stronger interaction between cationic liposomes and negatively charged bacterial membranes [61-63]. The *Salmonella* lysin BSP16Lys was encapsulated by a cationic liposome consisting of dipalmitoylphosphatidylcholine (DPPC), cholesterol and hexadecylamine. And its efficiency of passing through the OM of Gram-negative bacteria and activity to kill *Salmonella* and *E. coli* in the absence of treatment with an OM permeabilizer were increased [64].

Nanoparticles can also be used to encapsulate lysins. LysMR-5 was encapsulated by nanoparticles (Alg-Chi NPs) consisting of alginate and chitosan, resulting in enhanced bactericidal activity. The T4 lysozyme was coupled to cellulose nanocrystals (CNC) resulting in higher thermal stability and bactericidal activity against *E. coli* and *P. aeruginosa* compared to its free enzymes [65, 66]. Chitosan is a cationic polyelectrolyte with a number of advantageous biological properties, such as biodegradability, biocompatibility, low immunogenicity, non-toxicity, mucoadhesiveness and ability to increase membrane permeability [67]. Chitosan nanoparticles have also been well explored as macromolecular delivery vehicles such as peptides, proteins, nucleic acids and plasmids to augment their bioavailabilities as well as to protect them from biological environment, hence increasing their in-vivo half-life [68]. This encapsulation strategy using non-biohazardous and biocompatible particles such as chitosan can also be applied to the design of GN-lysins for food safety, such as the pneumocidal activity of endolysin Cpl-1 was maintained in chitosan nanoparticles [69].

### Combination with the OM Permeabilizers

The OM of Gram-negative bacteria effectively prevents most lysins from reaching the PG layer and acting on the cell wall externally. To increase the permeability of OM is one solution to solve this problem. Divalent cations (Mg^2+^ and Ca^2+^) are known to be crucial for the integrity of the bacterial OM. There are many kinds of OM permeabilizers which are generally divided into two categories. The first category is polyvalent cationic compounds such as polymyxin and its derivatives, aminoglycosides and lysine polymers. They can compete to replace the bivalent cations for the interaction with the anionic LPS molecules, leading to disorganization of the OM [70, 71].

The second category of OM permeabilizers is chelating agent. Chelation of divalent cations is a well-established method to permeabilize Gram-negative bacteria. Among numerous chelating agents, EDTA (ethylenediaminetetraacetic acid) is the most commonly used. EDTA removes divalent cations from their binding sites, causing OM disruption. Several studies had shown that EDTA had the strongest effect on cell wall penetration for lysins [15, 72, 73]. Weak organic acids in protonated form are also used as chelating agents. In general, most lysins are inactive at low pH, but the *Salmonella* phage endolysin Lys68 retained its protein structure and was enzymatically active in the pH range of 4.0-10.0, which was explained as the result of the combined action with weak organic acids (citric and malic) [74]. In fact, these weak organic acids were found to be membrane permeable at low pH. Further studies found that the permeabilizing effect of citric or malic acid on *Salmonella* infections was mainly due to acidification as like the effect of adding HCl at similar pH values.

OM permeabilizers could not only enhance the bactericidal activity of lysins, but could also broaden the lytic spectrum. The lysin ABgp46 only had the antibacterial activity against *A. baumannii* in the absence of OM permeabilizers. With the addition of citric and malic acids, its antibacterial activity against *A. baumannii* was enhanced and obtained lytic activities against *P. aeruginosa* and *S. typhimurium* [75].

## Conclusion

Bacteriophage endolysins are promising alternatives to antibiotics. The strategies that can be used to improve the enzyme properties (bactericidal activity, lysis spectrum, stability and targeting the substrate, etc) of bacteriophage endolysins are summarized as follows: Deletion of the cell wall-binding domain (For the CBD-independent lysins) or changing the net charge of catalytic domain; Addition of domains (Mainly addition of the cell wall-binding domain); DNA mutagenesis (Site-directed mutation and random mutagenesis); Chimerization of domains; Fusion to the MPPs (Helping lysins to reach the substrate); Fusion with domains targeting OM receptors or transport systems (Helping lysins to reach the substrate); Encapsulation strategy; Combination with the OM permeabilizers (Helping lysins to reach the substrate). Among these strategies, those related to adding other reagents in the enzyme reaction system, like encapsulation of lysins and the usage of OM permeabilizers, have to take into account the safety of those reagents, especially OM permeabilizers. The strategies used to improve GP-Lysins are also applicable to GN-Lysins and vice versa. It is also possible to explore the development of lysins with excellent performances by interoperability of multiple methods.

## Figures and Tables

**Table 1:** GN-Lysins that can pass through the outer membrane of Gram-negative bacteria.

Host bacteria	Lysins	References
*Acinetobacter baumannii*	LysAB3, LysAB4	[20]
	PlyAB1	[21]
	PlyF307	[22]
	Lys-ABP1-01	[23]
	Abgp46	[15]
	LysPA26	[24]
	LysAB54	[25]
*Pseudomonas aeruginosa*	LysPA26	[24]
*Cytrobacter fowleri*	CfP1	[26]
*Escherichia coli*	T5	[27]
	SPN9CC	[28]
